# Anterior Cingulate Cortex Activity During Rest Is Related to Alterations in Pain Perception in Aging

**DOI:** 10.3389/fnagi.2021.695200

**Published:** 2021-07-06

**Authors:** Juan L. Terrasa, Pedro Montoya, Carolina Sitges, Marian van der Meulen, Fernand Anton, Ana M. González-Roldán

**Affiliations:** ^1^Cognitive and Affective Neuroscience and Clinical Psychology, Research Institute of Health Sciences (IUNICS) and Balearic Islands Health Research Institute (IdISBa), University of the Balearic Islands (UIB), Palma, Spain; ^2^Institute for Health and Behavior, University of Luxembourg, Luxembourg, Luxembourg

**Keywords:** aging, pain, brain activity, EEG, fMRI, resting-state, anterior cingulate cortex

## Abstract

Alterations in the affective component of pain perception are related to the development of chronic pain and may contribute to the increased vulnerability to pain observed in aging. The present study analyzed age-related changes in resting-state brain activity and their possible relation to an increased pain perception in older adults. For this purpose, we compared EEG current source density and fMRI functional-connectivity at rest in older (*n* = 20, 66.21 ± 3.08 years) and younger adults (*n* = 21, 20.71 ± 2.30 years) and correlated those brain activity parameters with pain intensity and unpleasantness ratings elicited by painful stimulation. We found an age-related increase in beta2 and beta3 activity in temporal, frontal, and limbic areas, and a decrease in alpha activity in frontal areas. Moreover, older participants displayed increased functional connectivity in the anterior cingulate cortex (ACC) and the insula with precentral and postcentral gyrus. Finally, ACC beta3 activity was positively correlated with pain intensity and unpleasantness ratings in older, and ACC-precentral/postcentral gyrus connectivity was positively correlated with unpleasantness ratings in older and younger participants. These results reveal that ACC resting-state hyperactivity is a stable trait of brain aging and may underlie their characteristic altered pain perception.

## Introduction

Aging affects the functioning of pain-related neural networks, and therefore, the perception and modulation of pain (Farrell, 2012; Zhou and Shu, 2017; Cruz-Almeida et al., 2019; González-Roldán et al., 2020a). Nevertheless, the mechanisms of the increased pain perception described in older adults remain unclear. In a previous study, we examined the impact of age on cognitive pain modulation evoked by distraction in healthy older adults (González-Roldán et al., 2020b). We found that older participants experienced pain relief through distraction as did younger participants; however, they generally reported more pain in response to the painful stimulation. These results suggest that aging may enhance the affective assessment of pain perception. Alterations in the affective component of pain perception are related to the development of chronic pain (Baliki et al., 2008; Napadow et al., 2010; Bushnell et al., 2013) and could hence contribute to the increased vulnerability to chronic pain observed in older persons. In agreement, it has been suggested that the high prevalence of chronic pain in older adults (reaching over 50%) could be related to alterations in endogenous pain inhibition processes (Farrell, 2012; Lautenbacher et al., 2017) and/or in the affective component of the pain experience (Lautenbacher, 2012; Yezierski, 2012). Therefore, the importance of clarifying the brain mechanisms underlying this magnification in pain perception in older adults seems clear.

The study of brain activity during rest can be a promising approach to understand the neural underpinnings of pain alterations in aging. It has been shown that increased functional connectivity between primary and associative somatosensory areas during resting state is associated with the elevated pain thresholds that characterize older participants (González-Roldán et al., 2020a). Furthermore, resting-state functional networks, such as the default mode network (DMN) and the salience network, are altered in both healthy older individuals (Tomasi and Volkow, 2012; Huang et al., 2015) and chronic pain patients (Napadow et al., 2010; Kucyi and Davis, 2015; Martucci and Mackey, 2016). In this sense, it is well known that there is a strong relationship between resting-state functional connectivity (rsFC) of several brain areas belonging to these networks [i.e., anterior cingulate cortex (ACC), insula and amygdala] and pain perception in healthy young subjects (Proverbio et al., 2009; Ploner et al., 2010), as well as in patients with aging-related diseases such as Alzheimer’s disease (Beach et al., 2017). Thus, for instance, it has been shown that ACC baseline fluctuations positively correlate with pain intensity ratings after nociceptive stimulation in a healthy population (Boly et al., 2007). In agreement with fMRI results, EEG resting-state studies have shown a progressive change in the frequency and the distribution of brain waves during rest in aging (Vlahou et al., 2015; Ishii et al., 2017), most commonly in the form of alpha reductions (McEvoy et al., 2001; Babiloni et al., 2006; Rossini et al., 2007) and beta increments (Barry and De Blasio, 2017; Koelewijn et al., 2017).

Considering all these findings, the present study aimed to explore age-related changes in resting-state brain activity, and their possible relation to the increased pain perception described in older adults (Lautenbacher, 2012; Yezierski, 2012; González-Roldán et al., 2020b). For this purpose, pain intensity and pain unpleasantness ratings were correlated with EEG-based current density in several frequency bands and resting-state fMRI connectivity at rest. We hypothesized that older participants would show an increased beta and reduced alpha activity, as well as, a disturbed connectivity of the DMN and the salience networks. Moreover, we expected that the electrophysiological and hemodynamic alterations in pain-related areas, especially ACC (Proverbio et al., 2009; Ploner et al., 2010), would be related to changes in intensity and unpleasantness ratings of painful stimulation.

## Materials and Methods

### Participants and Procedure

Participants were recruited from the University of the Balearic Islands (the older group was recruited from a senior program). The sample was composed of 20 healthy older adults (nine men; 66.21 ± 3.08, age range of 60–72 years) and 21 healthy young adults (nine men; 20.71 ± 2.30, age range of 18–25 years). A complete sample description can be found in a previous study (González-Roldán et al., 2020b). In summary, none of the participants presented any psychiatric or neurological condition, history of drug abuse, cognitive impairment, acute or chronic pain, or left-handedness. Furthermore, both groups were comparable in depression, anxiety, and pain catrastrophizing scores. All participants were naive to the experiment and gave informed consent after the experimental procedure was explained. The study was conducted in accordance with the Declaration of Helsinki (1991) and was approved by the Ethics committee of the Balearic Islands (ref: IB 3429/13 PI).

The procedure was based on a single-blinded cross-sectional study. At laboratory arrival, participants completed a battery of questionnaires to assess their medical and psychological status (González-Roldán et al., 2020b). Then, participants underwent 7 min eyes closed resting-state EEG recording while comfortably seated in a quiet room. Afterward, electrical pain thresholds were assessed and the distraction task concomitant to a painful stimulation begun (see below a brief description of the task). Furthermore, all participants were invited to a resting-state fMRI session up to 4 months after the main experiment (mean days = 59.74 ± 31.15). Twelve young (six males) and 18 older adults (eight males) performed the fMRI session. The rest of the participants did not agree to participate (nine young participants) or did not fulfill the standard criteria for MRI recordings (one older participant).

### Painful Stimulation and Distraction Task

The experimental design of the painful stimulation under different levels of distraction to pain was reported in a previous study (González-Roldán et al., 2020b). In brief, painful electrical stimulation was presented to participants while they were performing a high distracting (1-back) or a low distracting (simple response) task. Electrical painful stimuli were delivered using a DS7A Digitimer device (Digitimer Limited—United Kingdom) and applied to the ventral side of the non-dominant wrist. The intensity of the pain stimulation used during the experiment was adapted to each participant’s individual pain threshold. Finally, subjects were asked to rate the intensity and unpleasantness of the painful stimulation with a computerized visual analog scale (VAS) under both high distracting and low distracting conditions. The VAS ranged from no pain/not unpleasant to worst pain imaginable/highly unpleasant.

### Brain Activity Acquisition and Analysis

#### Electrophysiological Recording and Analysis

All participants underwent an EEG resting-state recording for 7 min with their eyes closed. EEG signals were recorded using a QuickAmp amplifier (BrainProducts GmbH, Munich, Germany) from 60 scalp electrodes placed according to the international 10/20 system by using an average reference calculated by the amplifier. An electrooculogram (EOG) bipolar channel was obtained by placing one electrode above and another below the left eye. Electrode impedance was kept below 10 KΩ. EEG and EOG signals were recorded with a sampling rate of 1,000 Hz using BrainVision Recorder software (BrainProducts GmbH, Munich, Germany).

EEG recordings were further processed offline using BrainVision Analyzer (BrainProducts, Munich, Germany). A frequency bandpass filter of 0.1–30 Hz was applied. The first 2 min of the recording were discarded and therefore 5 min of resting-state activity were analyzed. EEG continuous data were segmented in epochs of 2,000 ms. Then, eye movement artifacts were corrected by the algorithm proposed by Gratton and Coles (Gratton et al., 1983). Thereafter, an artifact rejection protocol with the following criteria was applied: maximal allowed voltage step/sampling point = 100 mV, minimal allowed amplitude = −100 mV, maximal allowed amplitude = 100 mV, and maximal allowed absolute difference in the epoch = 100 mV. One older participant was excluded from the following analyses since the inclusion criteria were not met (less than 30% of the epochs rejected). To localize the cortical current density of the EEG resting-state activity, we used the exact low-resolution brain electromagnetic tomography, eLORETA software^1^ (Pascual-Marqui, 2002). It solves the so-called EEG inverse problem estimating “neural” current density values at any cortical voxel of the head volume conductor model. The brain model was based on a realistic cerebral shape partitioned in 6,239 voxels at 5 mm spatial resolution taken from a template of the Montreal Neurological Institute average MRI brain map (MNI152; Mazziotta et al., 2001). The current source density of the eLORETA image was calculated for each participant, within the following frequency bands: delta (1–4 Hz), theta (4–8 Hz), alpha (8–12 Hz), beta1 (12–16 Hz), beta2 (16–23 Hz), and beta3 (23–30 Hz). Subsequently, current source densities of all frequency bands during resting-state were estimated. To reduce inter-subject variability, spectra values were normalized at each voxel. Furthermore, a statistical non-parametric mapping randomization test was used to correct critical probability threshold values for multiple comparisons. A total of 5,000 permutations were used to determine the significance of each randomization test. Then, eLORETA current source densities of older and younger participants were compared for each frequency band by means of an independent-sample *t*-test. Finally, voxels with significant group differences (*p* < 0.05) were located in the MNI-brain and Brodmann areas (BA) to create the eLORETA images for each frequency band.

To investigate how group differences in source localization were related to altered pain responses, we correlated the current density of the most representative voxels showing significant differences between groups with the subjective pain intensity and unpleasantness ratings and pain thresholds obtained in the distraction task experiment for each condition (high and low distraction). For this purpose, Pearson correlations were computed separately for each group. After Bonferroni correction, the new *alpha* value was set at 0.01 (0.05/5).

#### MRI Acquisition and Analysis

fMRI data were collected with a GE 3 T scanner (General Electric Signa HDx, GE Healthcare, Milwaukee, WI) at the Son Espases University Hospital. For each participant, 240 whole-brain echo-planar images were acquired over 10 min with the eyes closed [36 transversal slices per volume; 3 mm slice thickness; 90 flip angles; repetition time (TR): 2,500 ms; echo time (TE): 35 ms; 64 × 64 matrix dimensions; 240 mm field of view; 3.75 × 3.75 × 3 mm voxel size]. The structural imaging data consisted of T1-weighted images. Data from 20 participants were acquired with the following parameters: 292 slices per volume; repetition time (TR): 7.84 s; echo time (TE): 2.976 ms; matrix dimensions, 256 × 256; 256 mm field of view; 1 mm slice thickness; 12 flip angles. The following parameters were used in 10 participants: 220 slices per volume; TR: 7.9 s; TE: 3 ms; matrix dimensions, 256 × 256; 256 mm field of view; 1 mm slice thickness; 12 flip angles. T1 imaging data was only used to perform intra-individual coregistration and nuisance pre-processing. Scanner noise was passively reduced by using in-ear hearing protection. Also, foam cushions were placed over the ears to restrict head motion and further to reduce the impact of scanner noise.

The analyses of functional connectivity at rest were performed with the CONN-fMRI fc toolbox v18a (Whitfield-Gabrieli and Nieto-Castanon, 2012) in conjunction with SPM12 (Wellcome Department of Imaging Neuroscience, London, UK^2^). All the structural and functional sequences were pre-processed using the CONN’s default pipeline for volume-based analysis following these steps: resampling to 2 × 2 × 2-mm voxels and unwarping, centering, slice time correction, normalization to the Montreal Neurological Institute (MNI) template, outlier detection (ART-based scrubbing), and smoothing to a 5-mm Gaussian kernel. Motion parameters were entered as multiple regressors and images with motion over 2.0 mm were regressed entirely out of the time course. Three participants from the older group (two men) had more than 20% of their images removed due to motion and were dropped from the analyses to ensure data quality. Furthermore, BOLD data underwent a denoising process by using the CompCor method (Behzadi et al., 2007) and applying a band-pass filter (0.01–0.09 Hz) in order to reduce both noise effects and low-frequency drift.

A Seed-to-Voxel functional connectivity analysis was then performed. Fifteen seeds of interest which are associated with the DMN and the salience networks were preselected from the Harvard-Oxford atlas. These regions were: medial prefrontal cortex (mPFC), bilateral lateral parietal cortex (LP), posterior cingulate cortex (PCC), ACC, bilateral insula (INS), bilateral rostral PFC, and bilateral supramarginal gyrus (SMG). Individual correlation maps were generated extracting the mean BOLD time course from the preselected seeds and calculating the correlation coefficients with the BOLD time-course of each voxel throughout the whole brain. We used an ANCOVA to examine group differences in Seed-to-Voxel connectivity using gender as a covariate. The threshold for significant changes was set to *p* < 0.05 whole-brain cluster level False Discovery Rate (FDR) corrected with a cluster building threshold of *p* < 0.001 uncorrected on a voxel level. Furthermore, a minimum cluster size of *k* > 100 voxels was also applied.

Finally, to investigate how group connectivity differences were related to altered pain responses, we correlated the rsFC of the seeds showing significant differences between groups with the subjective pain intensity and unpleasantness ratings obtained in the distraction task experiment for each condition (high and low distraction). Only the biggest resulting cluster for each seed was considered for correlational analyses. For this purpose, Pearson correlations were computed separately for each group. After Bonferroni correction, the new *alpha* value was set at 0.01 (0.05/5).

## Results

### Source Localization Results

**Figure 1:**
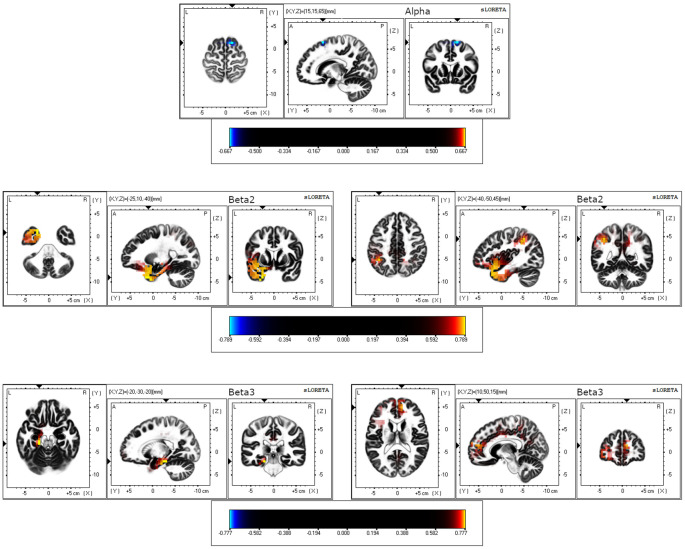
eLORETA results for three orthogonal brain slices (horizontal, sagittal, coronal) of alpha, beta2, and beta3 frequency bands. For alpha, the coordinate with the highest significance level is shown. For beta2 and beta3, the coordinates with the highest (left) and the second-highest (right) significant levels are displayed. Blue-colored voxels represent decreased (*p* < 0.05) current density in the older group compared to the younger group. Red/yellow-colored voxels represent increased (*p* < 0.05) current density in the older group compared to the younger group.

Differences between older and younger groups on statistical maps of source analyses for alpha, beta2, and beta3 resting-state activity are displayed in Table 1 and Figure 1. In the alpha band, analyses revealed a reduced current density in older adults in comparison to younger adults in the superior and middle frontal gyrus. In the beta2 band, analyses showed a generalized higher current density in the older group in comparison to the younger group in temporal (superior, middle, and inferior temporal gyrus), limbic [uncus, parahippocampal gyrus (PHG), ACC and PCC], parietal (precuneus and post-central gyrus), and frontal brain regions (pre-central gyrus and inferior, medial, middle, and superior frontal gyrus), as well as in the insula. Finally, in the beta3 band, the analyses yielded a significantly higher current density in the older group in comparison to the younger one in limbic (PHG, ACC, and PCC) and frontal regions (medial, middle, and superior frontal gyrus). All BA involved are shown in Table 1. No significant group differences were observed in other frequency bands.

**Table 1:** Summary of significant results from whole-brain eLORETA comparisons between older and younger groups for alpha, beta2, and beta3 frequency bands.

Lobe	Region	BA	X	Y	
**Alpha (Older < Younger)**					
Frontal	Superior frontal gyrus	6	15	15	65
		8	5	15	55
	Middle frontal gyrus	6	15	10	65
**Beta2 (Older > Younger)**					
Temporal	Superior temporal gyrus	38	−25	10	−40
		22	−50	15	−5
		13	−45	0	−10
		21	−50	−5	−10
		39	−35	−60	30
	Middle temporal gyrus	38	−35	5	−45
		21	−40	10	−40
	Inferior temporal gyrus	20	−30	0	−45
	Fusiform gyrus	20	−40	−10	−30
	Sub-Gyral	20	−40	−10	−25
Limbic	Uncus	38	−25	5	−45
		28	−20	5	−30
		20	−25	0	−45
		36	−20	0	−35
		34	−15	0	−25
	Parahippocampal gyrus	34	−20	5	−20
		28	−20	−10	−25
		35	−20	−10	−30
		36	−30	−20	−30
	Anterior cingulate	24	−5	−10	30
		32	15	35	20
		25	0	0	−5
	Posterior cingulate	31	15	−45	35
		23	−5	−40	25
		30	5	−45	20
		29	−5	−55	10
Parietal	Inferior parietal lobule	40	−40	−50	45
		39	−50	−65	40
	Supramarginal gyrus	40	−40	−45	35
	Precuneus	7	15	−50	40
		31	15	−50	35
	Postcentral gyrus	2	−40	−30	35
		3	−40	−25	40
	Angular gyrus	39	−55	−60	35
Frontal	Inferior frontal gyrus	47	−20	10	−20
		13	−30	15	−15
	Subcallosal gyrus	34	−25	5	−15
	Medial frontal gyrus	25	−15	10	−20
	Middle frontal gyrus	11	−40	35	−15
		47	−40	40	−5
		10	−40	55	−5
	Superior frontal gyrus	11	−30	45	−15
	Precentral gyrus	44	−45	10	10
		6	−55	0	10
		4	−55	−15	35
Sub-lobar	Insula	13	−35	10	−10
Occipital	Cuneus	18	0	−75	10
		19	−30	−85	30
	Superior occipital	19	−40	−85	30
**Beta3 (Older > Younger)**					
Limbic	Parahippocampal gyrus	35	−20	−30	−20
		28	−20	−20	−25
		36	−25	−30	−20
		34	−15	−10	−20
	Anterior cingulate	24	−5	−5	30
		32	10	45	10
		10	5	50	10
	Uncus	28	−15	−5	−30
		34	−15	−5	−25
	Posterior cingulate	23	−5	−15	30
Frontal	Medial frontal gyrus	10	10	50	15
		9	5	55	20
	Middle frontal gyrus	10	−40	55	−5
		11	−40	55	−10
	Superior frontal gyrus	10	10	65	20
		11	−30	60	−10
	Inferior frontal gyrus	10	−45	50	0

*Significant (*p* < 0.05) regions are indicated with the name of Brodmann area (BA) and MNI coordinates of the higher statistical two-tailed threshold (T) voxel*.

### Resting-State Functional Connectivity Results

**Figure 2:**
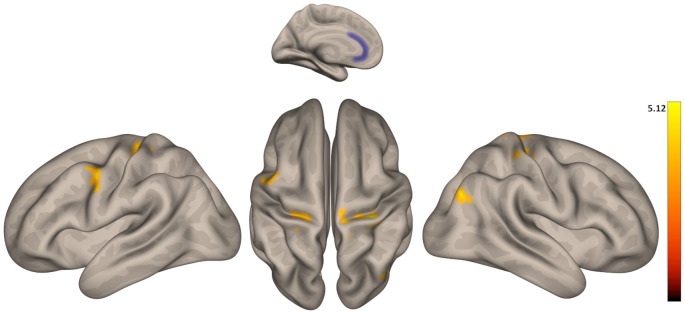
Group differences in functional connectivity between the anterior cingulate cortex (ACC) with precentral and postcentral gyrus. Color indicates the greater connectivity in older participants when compared with younger participants.

In general, functional connectivity of seeds from the DMN and salience networks was greater in older than in younger participants (Table 2). Regarding the DMN, we found greater connectivity in older vs. younger adults between LP (R) and inferior frontal gyrus; between LP (R), as well as the PCC, and a cluster including THA (R), putamen (R), and globus pallidus (R); between PCC and a cluster including the inferior frontal (R) and precentral gyrus (R); and between the PCC and the precentral gyrus (L). Regarding the salience network, we found greater connectivity in older vs. younger adults between ACC and bilateral pre- and post-central gyrus and ACC and superior occipital cortex (R; Figure 2); between INS (L) and: pre- and post-central gyrus (R), a cluster including posterior and temporooccipital middle temporal gyrus (L) and bilateral cuneus; between INS (R) and: postcentral gyrus (R), a cluster including precuneus and bilateral cuneus and a cluster including the putamen and globus pallidus (L). No areas with greater connectivity in younger vs. older adults were found.

**Table 2:** Resting-state functional connectivity between brain areas showing significant differences between groups.

			Older > Younger		
Seed	Cluster (x, y, z)	k	Cluster p-FDR	Peak *p*-unc	Result region
*DMN*
Lateral parietal cortex R	+24 +06 +10	315	0.000276	0.000003	Thalamus R
					Putamen R
					Pallidum R
	+52 +12 +20	141	0.018272	0.000002	Inferior frontal gyrus R
Posterior cingulate cortex	+38 +04 +26	462	0.000007	0.000004	Precentral gyrus R
					Inferior frontal gyrus R
	+16 −04 +12	221	0.001383	0.000004	Thalamus R
					Putamen R
					Pallidum R
	−44 +00 +24	144	0.009494	0.000004	Precentral gyrus L
*Salience*					
Anterior cingulate cortex	+32 −30 +56	291	0.000558	0.000001	Postcentral gyrus R
					Precentral gyrus R
	−50 +00 +44	165	0.009726	0.000018	Precentral gyrus L
	−18 −28 +58	138	0.015438	0.000037	Precentral gyrus L
					Postcentral gyrus L
	+40 −72 +30	115	0.025377	0.000006	Superior lateral occipital cortex R
Insula L	+32 −18 +60	249	0.001999	0.000001	Precentral gyrus R
					Postcentral gyrus R
	−48 −46 +02	152	0.018162	0.000000	Posterior middle temporal gyrus L
					Temporooccipital middle temporal gyrus L
	+00 −76 +30	131	0.024439	0.000006	Cuneal cortex L
					Cuneal cortex R
Insula R	+14 −42 +58	311	0.000373	0.000001	Postcentral gyrus R
	−02 −74 +32	265	0.000641	0.000001	Precuneous
					Cuneal cortex R
					Cuneal cortex L
	−28 −06 +04	125	0.032132	0.000000	Putamen L
					Pallidum L

### Correlational Analyses: Relationship Between Resting-State Brain Activity and Pain Perception

For correlational analyses of EEG data with subjective pain ratings, we selected the eight voxels from the different BAs with higher t-statistic value from regions showing a significant group difference in resting-state activity: superior frontal gyrus (BA 6) for alpha; superior temporal gyrus (BA 38), PCC (BA 31), inferior parietal lobule (BA 40), and insula (BA 13) for beta2; and PHG (BA 35), ACC (BA 24), and medial frontal gyrus (BA 10) for beta3. Results showed that beta3 activity at PHG (BA 35) and ACC (BA 24) were positively correlated with intensity and unpleasantness ratings in both distraction conditions (high and low) in the older participants. See Table 3 for *r* and *p* values. No other significant correlations were found neither in younger adults nor involving pain threshold scores.

**Table 3:** Pearson’s correlations between pain ratings and beta3 resting-state EEG current density in both groups.

	Beta3		
	PHG	Medial frontal	ACC
**Younger participants**			
Intensity-Low distraction	−0.168	−0.140	−0.398
Unpleasantness-Low distraction	−0.163	−0.024	−0.382
Intensity-High distraction	−0.154	−0.185	−0.271
Unpleasantness-High distraction	−0.069	−0.119	−0.205
**Older participants**			
Intensity-Low distraction	0.658**	0.526	0.637**
Unpleasantness-Low distraction	0.656**	0.515	0.588**
Intensity-High distraction	0.726***	0.437	0.686**
Unpleasantness-High distraction	0.666**	0.365	0.627**

R values are shown. Notice that the *p* value after the Bonferroni correction is equal 0.01. **p < 0.01, ***p < 0.001. PHG, parahippocampal gyrus; ACC, anterior cingulate cortex.

For correlational analyses of fMRI data with subjective pain ratings, we used all the rsFC values between the seeds and the resulting cluster showing the strongest statistical differences between groups. Results showed that ACC connectivity with the cluster including right pre- and post-central gyrus was positively correlated with unpleasantness ratings during both distraction conditions in older and younger adults, as well as, with pain intensity ratings during the low distraction condition in younger participants. See Table 4 for *r* and *p* values. No other significant correlations were found.

**Table 4:** Pearson’s correlations between pain ratings and resting-state functional connectivity in both groups.

Seed region	Insula L	Insula R	Lateral parietal cortex R	PCC	ACC
***Younger participants***					
Intensity-Low distraction	0.086	−0.071	0.517	0.456	0.732**
Unpleasantness-Low distraction	0.092	0.040	0.610	0.399	0.722**
Intensity-High distraction	0.253	0.078	0.396	0.384	0.662
Unpleasantness-High distraction	0.135	0.094	0.546	0.361	0.718**
***Older participants***					
Intensity-Low distraction	0.448	0.258	0.112	0.149	0.602
Unpleasantness-Low distraction	0.418	0.199	0.251	0.321	0.666**
Intensity-High distraction	0.421	0.150	0.022	0.092	0.538
Unpleasantness-High distraction	0.455	0.198	0.170	0.207	0.679**

R values are shown. Notice that the p value after the Bonferroni correction is equal 0.01. **p < 0.01. ACC, Anterior cingulate cortex; PCC, Posterior cingulate cortex; R, Right; L, Left.

## Discussion

In a previous study, we observed that older adults rated the pain as more intense and unpleasant than younger adults did, regardless of the context (high vs. low distraction) in which the nociceptive stimulation was applied (González-Roldán et al., 2020b). These results suggest that aging may enhance sensory and affective aspects of pain perception. In the present work, we explored the brain correlates of this pain perception enhancement in aging. For this purpose, we examined fMRI and EEG resting-state brain activity in older adults and correlated the possible alterations (in comparison to younger adults) with the previously mentioned pain ratings. Analyses of resting-state EEG data depicted that the older group showed increased resting-state current density of beta2 and beta3 activity mainly in temporal, frontal, and limbic areas, and decreased alpha activity in frontal areas in comparison to the younger group. Moreover, analyses of fMRI connectivity data showed that, in comparison to younger adults, older participants displayed increased rsFC between ACC and INS (from the salience network) and precentral and postcentral gyrus. Correlational analyses showed that, in older adults, PHG and ACC beta3 activity was positively associated with pain intensity and unpleasantness ratings in both distraction conditions; as well as, the ACC-precentral/postcentral gyrus rsFC was positively associated with unpleasantness ratings in both distraction conditions. Regarding young participants, ACC-precentral/postcentral gyrus rsFC was positively correlated with unpleasantness ratings during both distraction conditions, as well as, with pain intensity ratings in the low distraction condition.

Regarding the EEG alterations due to aging, we found that older participants showed a lower alpha activity focalized on the premotor cortex (superior and middle frontal gyrus) and widespread higher beta activity in several brain areas. A reduction of alpha activity related to aging is the most prominent electroencephalographic change in the brain (McEvoy et al., 2001; Babiloni et al., 2006; Rossini et al., 2007). Alpha waves are registered during normal wakeful state when the subject is quietly resting (Başar, 2012) and are proposed to represent a cortical “idling” that facilitates cortical activation by different stimuli (Pfurtscheller, 1992; Ishii et al., 2017). Furthermore, age-related alpha reduction has been associated with cognitive impairment in healthy and pathological aging (Babiloni et al., 2010, 2011). On the other hand, beta oscillations in the brain are commonly associated with movement-related activity and with attentional modulation processes (Honaga et al., 2010; Gola et al., 2013). We found increased beta oscillations in temporal, frontal, and limbic areas in older participants in comparison to younger participants. This result is in agreement with previous studies showing a widespread increased beta activity (Barry and De Blasio, 2017) or an increased beta band amplitude in somatosensory and parietofrontal resting-state networks (Koelewijn et al., 2017) in older adults compared to younger adults. Going beyond age-related changes in resting-state brain activity, we found that left PHG and ACC beta3 activity positively correlated with the pain intensity and unpleasantness ratings to painful stimulation. PHG is involved in pain modulation and sensitivity, and in anxiety-induced hyperalgesia (Ploghaus et al., 2001; Grant et al., 2010; Smallwood et al., 2013). ACC is activated in response to both acute and chronic pain (Zhuo, 2011; Bliss et al., 2016), and more specifically, seems to mediate the affective responses to noxious stimuli (Vogt, 2005; Xiao and Zhang, 2018). Furthermore, the ACC interacts with other cortical structures as part of the affective processing circuits and appears to play a role in pain-related unpleasantness and aversion (Bush et al., 2000; Wiech and Tracey, 2009). Therefore, the increase in beta3 resting-state activity in PHG and ACC reported in the present work supports the hypothesis of age-related alterations in the affective component of pain processing which is manifested in the augmented unpleasantness and intensity ratings to experimental pain.

Our imaging results also seem to support this conclusion. We found that older participants showed an increased rsFC compared to younger participants between ACC and INS (from the salience network) and somatosensory and motor areas as pre- and postcentral gyrus. More interestingly, rsFC between ACC and pre- and postcentral gyrus was positively correlated with the unpleasantness ratings in response to the pain stimulation in both high and low distraction conditions in the two groups of participants. Supporting our results, several studies have shown a strong relationship between rsFC of ACC and pain perception in healthy young subjects (Boly et al., 2007; Proverbio et al., 2009; Ploner et al., 2010) and in diseases such as Alzheimer’s (Beach et al., 2017). Furthermore, it seems that baseline fluctuations in ACC activity could be related to fluctuations in the subjective perception of identical nociceptive stimuli (Boly et al., 2007) and could predict individual pain thresholds (Spisak et al., 2020). Moreover, ACC and the salience network have been involved in attention to pain (Downar et al., 2003; Mouraux et al., 2011; Kucyi and Davis, 2015) and linked to the affective dimension of pain rather than to the sensory-discriminative dimension (Iwata et al., 2005; Xiao and Zhang, 2018). Therefore, the increased ACC-somatomotor rsFC in older subjects in comparison to younger subjects may suggest that aging is related to increases in the spontaneous communication of the ACC with somatomotor areas, which could lead to an increment in affective pain perception. This could also contribute to the larger vulnerability of older individuals in developing chronic pain (Leadley et al., 2012), given that increased connectivity between ACC and other pain-related brain areas has also been reported in several chronic pain conditions (Cifre et al., 2012; Schmidt-Wilcke, 2015; Martucci and Mackey, 2016; Malfliet et al., 2017). On the other hand, we found that older participants showed an increased rsFC compared to younger participants within the DMN, specifically between the LP cortex and the PCC, and regions involved in the sensory discriminative and affective motivational components of pain (Chudler and Dong, 1995; Ab Aziz and Ahmad, 2006; Yen and Lu, 2013), such as the thalamus and the basal ganglia. However, this change in rsFC was not related to pain perception.

It is important to mention that our study has a sample size limitation concerning the number of subjects yielding imaging data that merits consideration. However, previous studies using resting-state data have been published with similar sample sizes (Cole et al., 2006; Cifre et al., 2012; Beach et al., 2017). Furthermore, the high concordance between the EEG and fMRI analysis supports the reliability of our results. On the other hand, it is important to take into account that the distraction task may have affected the outcomes of pain perception. However, our results were consistent both in high and low distracting conditions. Further studies should replicate our findings, but we believe that our results contribute to a better understanding of the relationship between rsFC and pain in aging.

In summary, the present study revealed a resting-state hyperconnectivity of ACC in aging that seems to be associated with an age-related increase in affective pain ratings. Moreover, the fact that similar results were achieved with different brain imaging techniques (which indeed were recorded at different points in time), suggests that ACC hyperconnectivity could be a stable trait of brain aging. Future studies will be needed to explore if these alterations could be used as a biomarker for the predisposition of developing chronic pain in the older population and also be targeted by advanced techniques of brain modulation such as neurofeedback (Roy et al., 2020).

## Data Availability Statement

The datasets generated for this study are available on the request to the corresponding author.

## Ethics Statement

The studies involving human participants were reviewed and approved by Ethics Committee of the Balearic Islands (ref: IB 3429/17 PI). The patients/participants provided their written informed consent to participate in this study.

## Author Contributions

All authors discussed the results and commented on the manuscript. AG-R designed the study and did the data collection. JT and AG-R did the data analysis and wrote most of the manuscript but PM, CS, MM, and FA critically revised important parts of the manuscript. All authors contributed to the article and approved the submitted version.

## Conflict of Interest

The authors declare that the research was conducted in the absence of any commercial or financial relationships that could be construed as a potential conflict of interest.

## Footnotes

^1^http://arxiv.org/abs/0710.3341

^2^http://www.fil.ion.ucl.ac.uk/spm/

## References

[B1] Ab Aziz C. B. Ahmad A. H. (2006). The role of the thalamus in modulating pain. Malays. J. Med. Sci. 13, 11–18. 22589599 PMC3349479

[B2] Babiloni C. Binetti G. Cassarino A. Dal Forno G. Del Percio C. Ferreri F.. (2006). Sources of cortical rhythms in adults during physiological aging: a multicentric EEG study. Hum. Brain Mapp. 27, 162–172. 10.1002/hbm.20175 16108018 PMC6871339

[B3] Babiloni C. Lizio R. Vecchio F. Frisoni G. B. Pievani M. Geroldi C.. (2011). Reactivity of cortical alpha rhythms to eye opening in mild cognitive impairment and Alzheimer’s disease: an EEG study. J. Alzheimers Dis. 22, 1047–1064. 10.3233/JAD-2010-100798 20930306

[B4] Babiloni C. Visser P. J. Frisoni G. De Deyn P. P. Bresciani L. Jelic V.. (2010). Cortical sources of resting EEG rhythms in mild cognitive impairment and subjective memory complaint. Neurobiol. Aging 31, 1787–1798. 10.1016/j.neurobiolaging.2008.09.020 19027196

[B5] Baliki M. N. Geha P. Y. Apkarian A. V. Chialvo D. R. (2008). Beyond feeling: chronic pain hurts the brain, disrupting the default-mode network dynamics. J. Neurosci. 28, 1398–1403. 10.1523/JNEUROSCI.4123-07.2008 18256259 PMC6671589

[B6] Barry R. J. De Blasio F. M. (2017). EEG differences between eyes-closed and eyes-open resting remain in healthy ageing. Biol. Psychol. 129, 293–304. 10.1016/j.biopsycho.2017.09.010 28943465

[B7] Başar E. (2012). A review of alpha activity in integrative brain function: fundamental physiology, sensory coding, cognition and pathology. Int. J. Psychophysiol. 86, 1–24. 10.1097/01.NEP.0000000000000831 22820267

[B8] Beach P. A. Huck J. T. Zhu D. C. Bozoki A. C. Training D. O. Kuljiš R. O.. (2017). Altered behavioral and autonomic pain responses in Alzheimer’s disease are associated with dysfunctional affective, self-reflective and salience network resting-state connectivity. Front. Aging Neurosci. 9:297. 10.3389/fnagi.2017.00297 28959201 PMC5603705

[B9] Behzadi Y. Restom K. Liau J. Liu T. T. (2007). A component based noise correction method (CompCor) for BOLD and perfusion based fMRI. NeuroImage 37, 90–101. 10.1016/j.neuroimage.2007.04.042 17560126 PMC2214855

[B10] Bliss T. V. P. Collingridge G. L. Kaang B.-K. Zhuo M. (2016). Synaptic plasticity in the anterior cingulate cortex in acute and chronic pain. Nat. Rev. Neurosci. 17, 485–496. 10.1038/nrn.2016.68 27307118

[B11] Boly M. Balteau E. Schnakers C. Degueldre C. Moonen G. Luxen A.. (2007). Baseline brain activity fluctuations predict somatosensory perception in humans. Proc. Natl. Acad. Sci. 104, 12187–12192. 10.1073/pnas.0611404104 17616583 PMC1924544

[B12] Bush G. Luu P. Posner M. I. (2000). Cognitive and emotional influences in anterior cingulate cortex. Trends Cogn. Sci. 4, 215–222. 10.1016/s1364-6613(00)01483-2 10827444

[B13] Bushnell M. C. Čeko M. Low L. A. (2013). Cognitive and emotional control of pain and its disruption in chronic pain. Nat. Rev. Neurosci. 14, 502–511. 10.1038/nrn3516 23719569 PMC4465351

[B14] Chudler E. H. Dong W. K. (1995). The role of the basal ganglia in nociception and pain. Pain 60, 3–38. 10.1016/0304-3959(94)00172-B 7715939

[B15] Cifre I. Sitges C. Fraiman D. Muñoz M. Á. Balenzuela P. González-Roldán A.. (2012). Disrupted functional connectivity of the pain network in fibromyalgia. Psychosom. Med. 74, 55–62. 10.1097/PSY.0b013e3182408f04 22210242

[B16] Cole L. J. Farrell M. J. Duff E. P. Barber J. B. Egan G. F. Gibson S. J. (2006). Pain sensitivity and fMRI pain-related brain activity in Alzheimer’s disease. Brain 129, 2957–2965. 10.1093/brain/awl228 16951408

[B17] Cruz-Almeida Y. Fillingim R. B. Riley J. L. Woods A. J. Porges E. Cohen R.. (2019). Chronic pain is associated with a brain aging biomarker in community-dwelling older adults. Pain 160, 1119–1130. 10.1097/j.pain.0000000000001491 31009418 PMC6752890

[B18] Downar J. Mikulis D. J. Davis K. D. (2003). Neural correlates of the prolonged salience of painful stimulation. NeuroImage 20, 1540–1551. 10.1016/s1053-8119(03)00407-5 14642466

[B19] Farrell M. J. (2012). Age-related changes in the structure and function of brain regions involved in pain processing. Pain Med. 13, S37–S43. 10.1111/j.1526-4637.2011.01287.x 22497746

[B20] Gola M. Magnuski M. Szumska I. Wróbel A. (2013). EEG beta band activity is related to attention and attentional deficits in the visual performance of elderly subjects. Int. J. Psychophysiol. 89, 334–341. 10.1016/j.ijpsycho.2013.05.007 23688673

[B21] González-Roldán A. M. Terrasa J. L. Sitges C. van der Meulen M. Anton F. Montoya P. (2020a). Age-related changes in pain perception are associated with altered functional connectivity during resting state. Front. Aging Neurosci. 12:116. 10.3389/fnagi.2020.00116 32457594 PMC7221150

[B22] González-Roldán A. M. Terrasa J. L. Sitges C. van der Meulen M. Anton F. Montoya P. (2020b). Alterations in neural responses and pain perception in older adults during distraction. Psychosom. Med. 82, 869–876. 10.1097/PSY.0000000000000870 33003073

[B23] Grant J. A. Courtemanche J. Duerden E. G. Duncan G. H. Rainville P. (2010). Cortical thickness and pain sensitivity in zen meditators. Emotion 10, 43–53. 10.1037/a0018334 20141301

[B24] Gratton G. Coles M. G. H. Donchin E. (1983). A new method for off-line removal of ocular artifact. Electroencephalogr. Clin. Neurophysiol. 55, 468–484. 10.1016/0013-4694(83)90135-9 6187540

[B25] Honaga E. Ishii R. Kurimoto R. Canuet L. Ikezawa K. Takahashi H.. (2010). Post-movement beta rebound abnormality as indicator of mirror neuron system dysfunction in autistic spectrum disorder: an MEG study. Neurosci. Lett. 478, 141–145. 10.1016/j.neulet.2010.05.004 20452402

[B26] Huang C.-C. Hsieh W.-J. Lee P.-L. Peng L.-N. Liu L.-K. Lee W.-J.. (2015). Age-related changes in resting-state networks of a large sample size of healthy elderly. CNS Neurosci. Ther. 21, 817–825. 10.1111/cns.12396 25864728 PMC6493082

[B27] Ishii R. Canuet L. Aoki Y. Hata M. Iwase M. Ikeda S.. (2017). Healthy and pathological brain aging: from the perspective of oscillations, functional connectivity and signal complexity. Neuropsychobiology 75, 151–161. 10.1159/000486870 29466802

[B28] Iwata K. Kamo H. Ogawa A. Tsuboi Y. Noma N. Mitsuhashi Y.. (2005). Anterior cingulate cortical neuronal activity during perception of noxious thermal stimuli in monkeys. J. Neurophysiol. 94, 1980–1991. 10.1152/jn.00190.2005 15928063

[B29] Koelewijn L. Bompas A. Tales A. Brookes M. J. Muthukumaraswamy S. D. Bayer A.. (2017). Alzheimer’s disease disrupts alpha and beta-band resting-state oscillatory network connectivity. Clin. Neurophysiol. 128, 2347–2357. 10.1016/j.clinph.2017.04.018 28571910 PMC5674981

[B30] Kucyi A. Davis K. D. (2015). The dynamic pain connectome. Trends Neurosci. 38, 86–95. 10.1016/j.tins.2014.11.006 25541287

[B31] Lautenbacher S. (2012). Experimental approaches in the study of pain in the elderly. Pain Med. 13, S44–S50. 10.1111/j.1526-4637.2012.01326.x 22497747

[B32] Lautenbacher S. Peters J. H. Heesen M. Scheel J. Kunz M. (2017). Age changes in pain perception: a systematic-review and meta-analysis of age effects on pain and tolerance thresholds. Neurosci. Biobehav. Rev. 75, 104–113. 10.1016/j.neubiorev.2017.01.039 28159611

[B33] Leadley R. M. Armstrong N. Lee Y. C. Allen A. Kleijnen J. (2012). Chronic diseases in the european union: the prevalence and health cost implications of chronic pain. J. Pain Palliat. Care Pharmacother. 26, 310–325. 10.3109/15360288.2012.736933 23216170

[B34] Malfliet A. Coppieters I. Van Wilgen P. Kregel J. De Pauw R. Dolphens M.. (2017). Brain changes associated with cognitive and emotional factors in chronic pain: a systematic review. Eur. J. Pain 21, 769–786. 10.1002/ejp.1003 28146315

[B35] Martucci K. T. Mackey S. C. (2016). Imaging pain. Anesthesiol. Clin. 34, 255–269. 10.1016/j.anclin.2016.01.001 27208709 PMC5289642

[B36] Mazziotta J. Toga A. Evans A. Fox P. Lancaster J. Zilles K.. (2001). A probabilistic atlas and reference system for the human brain international consortium for brain mapping (ICBM). Philos. Trans. R. Soc. Lond. B. Biol. Sci. 356, 1293–1322. 10.1098/rstb.2001.0915 11545704 PMC1088516

[B37] McEvoy L. K. Pellouchoud E. Smith M. E. Gevins A. (2001). Neurophysiological signals of working memory in normal aging. Cogn. Brain Res. 11, 363–376. 10.1016/s0926-6410(01)00009-x 11339986

[B38] Mouraux A. Diukova A. Lee M. C. Wise R. G. Iannetti G. D. (2011). A multisensory investigation of the functional significance of the “pain matrix”. NeuroImage 54, 2237–2249. 10.1016/j.neuroimage.2010.09.084 20932917

[B39] Napadow V. LaCount L. Park K. As-Sanie S. Clauw D. J. Harris R. E. (2010). Intrinsic brain connectivity in fibromyalgia is associated with chronic pain intensity. Arthritis Rheum. 62, 2545–2555. 10.1002/art.27497 20506181 PMC2921024

[B40] Pascual-Marqui R. D. (2002). Standardized low-resolution brain electromagnetic tomography (sLORETA): technical details. Methods Find. Exp. Clin. Pharmacol. 24, 5–12. 12575463

[B41] Pfurtscheller G. (1992). Event-related synchronization (ERS): an electrophysiological correlate of cortical areas at rest. Electroencephalogr. Clin. Neurophysiol. 83, 62–69. 10.1016/0013-4694(92)90133-3 1376667

[B42] Ploghaus A. Narain C. Beckmann C. F. Clare S. Bantick S. Wise R.. (2001). Exacerbation of pain by anxiety is associated with activity in a hippocampal network. J. Neurosci. 21, 9896–9903. 10.1523/JNEUROSCI.21-24-09896.2001 11739597 PMC6763058

[B43] Ploner M. Lee M. C. Wiech K. Bingel U. Tracey I. (2010). Prestimulus functional connectivity determines pain perception in humans. Proc. Natl. Acad. Sci. 107, 355–360. 10.1073/pnas.0906186106 19948949 PMC2806712

[B44] Proverbio A. M. Adorni R. Zani A. Trestianu L. (2009). Sex differences in the brain response to affective scenes with or without humans. Neuropsychologia 47, 2374–2388. 10.1038/s41586-021-03681-2 19061906

[B45] Rossini P. M. Rossi S. Babiloni C. Polich J. (2007). Clinical neurophysiology of aging brain: from normal aging to neurodegeneration. Prog. Neurobiol. 83, 375–400. 10.1016/j.pneurobio.2007.07.010 17870229

[B46] Roy R. de la Vega R. Jensen M. P. Miró J. (2020). Neurofeedback for pain management: a systematic review. Front. Neurosci. 14:671. 10.3389/fnins.2020.00671 32765208 PMC7378966

[B47] Schmidt-Wilcke T. (2015). Neuroimaging of chronic pain. Best Pract. Res. Clin. Rheumatol. 29, 29–41. 10.1016/j.berh.2015.04.030 26266997

[B48] Smallwood R. F. Laird A. R. Ramage A. E. Parkinson A. L. Lewis J. Clauw D. J.. (2013). Structural brain anomalies and chronic pain: a quantitative meta-analysis of gray matter volume. J. Pain 14, 663–675. 10.1016/j.jpain.2013.03.001 23685185 PMC4827858

[B49] Spisak T. Kincses B. Schlitt F. Zunhammer M. Schmidt-Wilcke T. Kincses Z. T.. (2020). Pain-free resting-state functional brain connectivity predicts individual pain sensitivity. Nat. Commun. 11:187. 10.1038/s41467-019-13785-z 31924769 PMC6954277

[B50] Tomasi D. Volkow N. D. (2012). Aging and functional brain networks. Mol. Psychiatry 17, 549–558. 10.1038/mp.2011.81 PMC3193908 21727896

[B51] Vlahou E. L. Thurm F. Kolassa I.-T. Schlee W. (2015). Resting-state slow wave power, healthy aging and cognitive performance. Sci. Rep. 4:5101. 10.1038/srep05101 24869503 PMC4037748

[B52] Vogt B. A. (2005). Pain and emotion interactions in subregions of the cingulate gyrus. Nat. Rev. Neurosci. 6, 533–544. 10.1038/nrn1704 15995724 PMC2659949

[B53] Whitfield-Gabrieli S. Nieto-Castanon A. (2012). Conn: a functional connectivity toolbox for correlated and anticorrelated brain networks. Brain Connect. 2, 125–141. 10.1089/brain.2012.0073 22642651

[B54] Wiech K. Tracey I. (2009). The influence of negative emotions on pain: Behavioral effects and neural mechanisms. NeuroImage 47, 987–994. 10.1016/j.neuroimage.2009.05.059 19481610

[B55] Xiao X. Zhang Y.-Q. (2018). A new perspective on the anterior cingulate cortex and affective pain. Neurosci. Biobehav. Rev. 90, 200–211. 10.1016/j.neubiorev.2018.03.022 29698697

[B56] Yen C.-T. Lu P.-L. (2013). Thalamus and pain. Acta Anaesthesiol. Taiwan 51, 73–80. 10.1016/j.aat.2013.06.011 23968658

[B57] Yezierski R. P. (2012). The effects of age on pain sensitivity: preclinical studies. Pain Med. 13, S27–S36. 10.1111/j.1526-4637.2011.01311.x 22497745 PMC3565621

[B58] Zhou W. Shu H. (2017). A meta-analysis of functional magnetic resonance imaging studies of eye movements and visual word reading. Brain Behav. 7:e00683. 10.1002/brb3.683 28523225 PMC5434188

[B59] Zhuo M. (2011). Cortical Plasticity as a New Endpoint Measurement for Chronic Pain. Mol. Pain 7:54. 10.1186/1744-8069-7-54 21798042 PMC3157449

